# Automatic analysis: the laboratory manager's problems

**DOI:** 10.1155/S1463924680000041

**Published:** 1980

**Authors:** J. K. Foreman

**Affiliations:** National Physical Laboratory Teddington Middlesex UK


### Automatic analysis: the laboratory manager's problems

J.K. Foreman
*National Physical Laboratory, Teddington, Middlesex, U.K.*

### Introduction

Automation of analytical methods and the introduction of automation into laboratories is growing at an ever-increasing pace and very few laboratory managers will escape the task of bringing some measure of automation into their own laboratory. This text is aimed primarily at the manager contemplating, or being urged to contemplate, automating certain of his currently manually operated procedures. It may also have some value for the manager already familiar with automated methods who wishes to incorporate more extensive automation or adopt second generation automated procedures, in which microelectronic and computer technology are more closely integrated with automated chemistry. To the uninvolved observer the introduction of automation may appear to constitute little more than abandoning glassware and manually operated instruments for an impressive array of well designed and engineered plastic and metal boxes which apparently fulfil the same purpose in return for a reduction in human effort. But experience to date [1. 2] supports the more enlightened viewpoint that introducing a substantial amount of automatic equipment alters the 'life style' of the laboratory and its staff in several fundamental and far-reaching ways and that the laboratory manager cannot stand aside from these changes; he must adapt to them at an early stage and preferably play the leading role in encouraging and planning their introduction. This paper is concerned with these changes, the background and reasons for them and it attempts to offer some general guidance for coming to terms with automation.

The ramifications of introducing automated procedures reach far beyond the acceptance of new lamps for old and the successful adaptation to the resulting change requires a broad-based and forward-looking approach on the manager's part, together with a capability for self-appraisal and self-education. One thing is certain, if a good case exists for transferring from a manual to an automatic regime, then properly selected automatic equipment will bring benefits and advantages far in excess over a few inevitable, but generally soluble, shortcomings arising from the adaptation to a changed working discipline. So much depends on the nature and strength of the case for automation and this is related to the type and size of the analytical facility, its role in the overall organization and the customers for its services, in particular whether the latter are part of the same organization or external to it. The former case is by far the more common and is represented by the analytical laboratories of manufacturing and processing industries, of supply industries (water, power etc) and of hospitals. Consultant and independent testing laboratories fall into the second category.

Broadly speaking, for a laboratory engaged in providing analysis as a service, the motivation to automated methods is predominantly an economic one, either to reduce the cost per sample analysis, to improve turn-round time, to increase analytical capacity or to release experienced staff for more complex or demanding problems.

**Table 1. Abbreviated representation of responsibilities of a laboratory manager**

1. Managing the laboratory: its contents, workload, safety and security
2. Managing laboratory staff: responsibility for staff performance, morale, career development, education and training
3. Responsibility to customer(s) for laboratory output

### The laboratory manager's responsibilities

Realistic economic evaluation of the change from manual to automatic analysis is a critical responsibility of the laboratory manager. But in addition the availability of suitably qualified and trained operating staff and the need to maintain a close rapport with the customer are of significant interest. These and other aspects are discussed below. Before looking at these it is helpful to have a simple representation of an analytical laboratory manager's responsibilities; of the many ways of expressing and classifying these the statement in Table 1 has been selected. It illustrates the functions in terms of animate and inanimate (people and things) responsibilities. In practice these functions interact and often overlap to some degree. Furthermore the relative emphasis on each category is variable depending on the size and role of the laboratory, the complexity of the workload, the number, quality and experience of the staff and the standard of support facilities available. Nevertheless Table 1 constitutes a generic guide and a logical pattern for discussion.

### The case of automation

The manager is concerned with the adequacy of laboratory techniques and equipment to meet the analytical demands placed on the laboratory. The relationships between method performance and customer requirement, optimum utilization of the various grades of laboratory staff and overall cost effectiveness of the laboratory are typically the criteria against which laboratory performance must be evaluated.The initial thoughts of introducing a measure of automation usually arise from these assessments though on occasion they may be prompted by acute shortage of properly qualified and trained personnel. However, for the reasons noted in the preceding section the overwhelming motivation for introducing automated procedures is an economic one.

Whatever the grounds for contemplating automation its introduction involves a financial investment, directly in terms of purchase of equipment and, possibly, indirectly through ancillary costs of structural modifications to the laboratory, education and training of scientific staff and, if large volume automation is contemplated, through changes in the organization of processing, disseminating and storage of results. Unless the laboratory manager is totally self-financing, as in the case of the principal of a consultancy, he must start by preparing a convincing case to top management for the expenditure, and because many factors, often inter-related, influence the argument, even the fully independent manager would be unwise to omit this important exercise of justification. A cost-benefit analysis must constitute the major platform of the case. This analysis must start with a specification of what the analytical laboratory is required to do, taking cognizance of any real constraints, for example the analytical result might be required rapidly either to reduce storage demands in production areas or to prevent any harm to patients in distress in a clinical environment. The specification should be comprehensive in terms of current and foreseeable tasks including any ancillary benefits which may arise from the availability of automatic equipment, for example improved facilities for data manipulation. These could facilitate the production of valuable additional information like detailed statistical analyses, which may be beyond the reach of a laboratory equipped solely for manual analysis.

A first-order cost comparison between manual and automatic execution of the task comprising the specification can be made by calculating the cost of the manual approach and the capital, operating and likely maintenance cost of the automatic approach over a period of time allowed for depreciation of the capital investment. The principles for conducting this cost comparison have been presented in detail elsewhere [3] and are not repeated here. Nevertheless several important factors are worthy of brief mention. A simple comparison of cost per sample analysis by manual and automatic means does not tell the full story. Automatic techniques unlike manual ones, rarely demand full-time operator attendance and the potential value of staff time so released must be taken into account. Automatic analyzers offer the possibility of round-the-clock operation and the economic attractiveness of this must be weighed against the additional penalties incurred by the improved safety and security requirements. A judgment regarding long term reliability of automatic equipment is also implied. A further factor for consideration is whether the conversion to automatic analysis is to be complete or whether a degree of manual back-up is to be retained. Certain designs of automatic analyzer are such that a malfunction can cause the irrevocable loss of results on a series of samples; to what extent can this be accommodated? Considerations of this type influence the choice of automatic equipment.

It must also be borne in mind that the internal costing as described above may be over-ridden by the greater savings which automatic analysis can bring to other parts of the organization to which the laboratory belongs. A common instance occurs in large scale processing or production plants; products often cannot be released for distribution nor intermediates passed to the next stage of the process, until receipt of analytical evidence that the material conforms to specification. In a production plant this implies the provision of delay storage vessels, the cost of which is likely to far exceed the cost of the automatic analytical equipment. In most, though not all, cases automatic analysis results in faster analysis which in turn reduces the demand for costly storage facilities. This could be a prime consideration in the design of new plant.

Other aspects of the case for which the laboratory manager must provide information include possible changes in staffing requirements; typically the introduction of automation can lead to some reduction in overall complement but this can be partially offset by the need to ensure the availability of staff skilled in instrumental, especially mechanical and electronic, techniques to maintain operation and correct any malfunction of the automatic equipment.

The items mentioned above for evaluation in presenting the case for automation are by no means exhaustive. Special considerations and constraints are likely to arise in each laboratory manager's sphere of responsibility and these will need to be assessed on a cost-benefit and customer-benefit basis and included in the total analysis.

The complete case for automation must also include recommendations as to how the decision to automate should be implemented. This can be achieved by purchase of commercial equipment and if this course is favoured then the type must be specified. Alternatively the proposal may be to purchase commercial equipment and modify it to achieve the desired configuration, if so the nature of the modifications must be clearly stated together with the proposed means of carrying them out, whether by the maker or by using the laboratory's or organization's own resources. Finally a recommendation may be made to design and construct the automatic equipment in-house, in which case a design specification is required, supported by evidence that the organization possesses, or has access to, the necessary engineering support [4].

Whatever the case for contemplating automation, the presentation and evaluation of the evidence requires judgements on the part of the laboratory manager; unless the manager already has considerable experience of automatic analysis and its ramifications, he will be in no position to make these judgments in isolation. The manager lacking this experience must embark on a process of self education by means such as first hand discussions with those whose knowledge can assist him, for example manufacturers of automatic equipment and experienced users of it. He should also discuss the problems with his own staff as an aid to identifying potential operational problems and, above all, maintain a full and regular dialogue with the "customers" for his analyses to ensure that the automatic regime will continue to provide the service required and that his plans in no respect compromise the "customer's" requirements for the way in which information is presented. It is indeed likely that a well-conceived automatic procedure will be able to offer the customer an improved or more extensive service

### Implementation

Closely parallel to the development of the case for installing automatic equipment is the means by which, if approved, the scheme will be implemented. It is assumed that the laboratory manager will be responsible for the implementation though it is recognized that this may not necessarily be so. But even if the implementation responsibility falls elsewhere it is highly desirable that whoever is involved should be fully aware of the initial thinking and of the factors influencing choice of instrumentation. The first stage of implementation, whether to buy commercial automatic equipment with or without modification, or whether to design and construct the necessary instrumentation has been mentioned above as an integral part of the case. But once this decision is made many more detailed issues need to be resolved, indeed some of them may need prior consideration because they can contribute to the decision process. These are briefly discussed below and again they emphasise the need for the manager to become involved in both the scientific and organizational aspects of automatic analysis.

If it is concluded that the analytical requirements are such that they can be met by purchasing commercial equipment, then the manager must make a detailed appraisal of the many design options available. Although they are not mutually exclusive there are two well-defined approaches to automating chemical analyses; the discrete approach, in which each sample is maintained as a separate entity throughout the analysis and is transported to individual stations for operations like dilution, reagent addition, incubation and measurement, and the continuous method, wherein each sample is converted to a flowing stream and reactions are carried out by merging streams, the final measurement being made in a flow-through unit. The advantages and disadvantages of discrete and continuous methods are summarized elsewhere [5] but the choice of approach usually rests on either the complexity of the analysis, the sample throughput or the relative capital and maintenance costs. Recently, variants of both approaches have become commercially available. Centrifugal analysis is a compact, rapid discrete technique and flow-injection analysis is an inherently simple and inexpensive continuous flow method. The literature references to both approaches is rapidly increasing and though the range of proven applications is currently rather limited, they both merit consideration as potential sol-

**Table 2. Principal stages in the development of a prototype automatic analyzer**

1. Specification of (a) the problem
   (b) the possible solution
2. System analysis and feasibility study
3. Prototype design
4. Construction
5. Installation and testing
6. Assessment of performance utions to a number of analytical problems. The manager must also decide whether the chemical (and physical) stages of the manual method are all amenable to automation. Certain separation procedures, notably precipitation and filtration, although available in a number of automatic analysers, tend to be less convenient and reliable than for example automatic solvent extraction. Indeed the manager should submit each of his manual methods to this type of scrutiny and, where appropriate, consider alternative analytical methods offering greater compatibility with the automatic analyzer. It is critically important, both from operational and cost effectiveness standpoints, to match the complexity of the purchased equipment to the analytical requirement. Many manufacturers offer a range of options over and above the basic instrument, notably in respect of computing and data handling facilities. Whilst the manager should assure himself that the package proposed for purchase will meet all aspects of his analytical programme he must also resist the temptation to acquire sophisticated and often expensive extras which he has only the remotest prospect of using. Equally, however, under-provision must be avoided because it inevitably leads to frustration both for laboratory personnel and the customer. The correct judgment can only be derived through a detailed evaluation of current and envisaged commitments in terms of sample load, sample variety and method performance.

If the manager recommends that automatic analytical equipment should be designed and constructed to meet his needs, then he will almost certainly be involved in many decisions, most of them of a specialized nature. Generally speaking a decision to design and construct rather than purchase, implies that no commercially available equipment, with or without modification, can adequately meet the analytical needs. Certainly any economic evaluation would favour purchase rather than construction if the former option is a valid one. Embarking upon the design and construction route also carries the implicit assumption that the organization for which the manager provides the analytical service possesses the range of engineering and workshop disciplines to implement the decision or has access to them. In either event the responsibility for carrying out much of the work will pass from him to others in the engineering disciplines. Nevertheless he retains the all-important responsibility for ensuring that those involved in the engineering work are fully and correctly informed on all aspects of the project. Indeed the quality of the rapport between the user and maker of the equipment has a profound influence on the extent to which the venture is successful. Unnecessary modifications at all stages of the design and fabrication procedure must be avoided by effective liaison. Such liaison should also ensure maximum cost-effectiveness and circumvent any delays in completion of the equipment.

The principal stages in the development of an automatic analyzer to the prototype stage are listed in Table 2. The extent to which the laboratory manager needs to be involved in the various stages is largely self-evident. Porter and Stockwell [4] have discussed the issues in detail and comment here is limited to a few salient points. The specification is critically important; an effective design and final product is utterly dependent upon a full and accurate specification of the need. The equipment must achieve the desired performance at each stage of the method and therefore the manager must specify the method and its anticipated performance in detail. He must, jointly with the designers, attempt to achieve maximum compatibility between the basic chemical operations and also engineering design procedures. This will usually require experimental support through research and development studies in the chemical laboratory. Unless the manager has engineering skills his involvement at the prototype design stage will be less, and the design engineer, armed with the specification provided by the manager, will take the lead. But the laboratory manager must retain close contact with the design team and, within the limits of his capability, seek to ensure that the design is in accord with the specified requirements. In particular he must assure himself that the recommended materials of construction are capable of the desired performance and that leakage, wear, and corrosion are not likely to arise. He should pay special attention to the provisions made for adequacy and ease of maintenance. The construction stage will also be primarily a responsibility of the engineering staff but again the laboratory manager can contribute to the effectiveness of the project. To take but one example, construction entails testing of components and units; whilst many of these will be bench tests of a purely mechanical or electronic nature some will require laboratory evaluation. The manager should ensure that he and his analytical team participate fully in these evaluations and in the subsequent assessments of the instrument performance. Such testing frequently reveals design faults and limitations and it is prudent to correct or eradicate these at the earliest opportunity. Once the prototype is complete the laboratory manager assumes responsibility for installing it and testing it against his initial specification. In performing the tests and evaluating the results he should work closely with the design and construction staff, who must remain closely involved not only to correct any inadequacies in the performance of the prototype but also to finalize design data and instrument manuals for the finished product.

Experience shows that the complete process of specifying, designing and fabricating an automatic analyzer and also implementing it successfully in a routine environment is a complex task, involving many stages and a number of interactions between staff. Although, as stated above, the laboratory manager does not play the leading role in all of it he is nevertheless a key figure throughout. By ensuring that a continuing and constructive dialogue is maintained between the engineering staff and those of his own laboratory, he can ensure that potential problems are recognised and acted upon quickly, but can also maintain a high level of mutual confidence in the ultimate success of the project.. There is nothing more damaging to the success of a project than a lack of confidence of credibility between the groups involved. Setbacks in any development project are virtually inevitable but these can be effectively overcome as long as confidence in the viability of the project is maintained.

When the automatic analyzer has be purchased or constructed the manager will arrange for it to be installed in his laboratory and thereafter he will be responsible for planning, performing and assessing the necessary commissioning trials and for the subsequent operation of the equipment. Operation includes attention to relevant safety aspects and good preventative maintenance. At this point the manager's animate responsibilities, which are dealt with specifically in the following section, become clearly evident. While the enlightened manager will have involved both his staff and customers throughout the stages prior to the acquisition of the automatic equipment, once it is operational both staff and customers become directly and specifically concerned. The principal stages in any analytical procedure are listed in Table 3.

In transferring from manual to automatic analysis, the regime for one or more of these stages will change and the working discipline of both the laboratory manager and his staff must be adjusted, not only to meet the new operational conditions but to achieve an effective and economic mode of working. The manager must evolve a method of working which utilizes the analyzer effectively and also makes best use of the varied talents of the staff available. For this he will use experience gained, and he must be prepared to modify the work patterns on a continuing basis especially in relation to trouble-shooting rectification of faults. Referring to the list in Table 3 the sampling procedure may or may not change; if the introduction of automatic analysis allows a large increase in sample throughput it is likely that any change in sampling regime will occur external to the analytical laboratory. Any large increase in the rate of processing of samples will necessitate a review of the methods for recording analytical data and the manner in which data are processed. Automatic calculation and reporting using computers, or increasingly microcomputers, is almost certain to replace manual calculations; this can be achieved in many ways but the general aim is to use a single processor to accept data and produce the required analytical information by performing the necessary computations. The approach chosen will depend on the volume of data to be processed, and on the complexity of the calculation generating the information required by the customer. On no account should the laboratory manager authorize a change in the reporting layout without first consulting the customer. The latter may readily accept results in direct print-out form, but it is an over-riding principle that the analyst should provide results in the most readily assimilable form for subsequent use. Designing a computer-produced report form to the customer's specification is rarely a major problem, but often not adequately covered.

When automatic systems are implemented, one further aspect with which the laboratory manager must concern himself is calibration. It is important that he introduces an appropriate standardization programme, and for the manager to assure himself of the adequacy and representativeness of his calibration standards. This can pose serious problems especially when it is difficult to guarantee that the chemical form of the analyte is the same in both sample and standard.

### Staff matters

A crucial responsibility of the laboratory manager is to prepare his staff for any changes in working method that result from the introduction of automatic analysis and to guide them through the adaptation process. It is normal, and understandable, for a skilled and experience analyst to feel that much of his skill and pride has been replaced by management in favour of a machine incapable of thought. But the analytical responsibilities have been modified and not reduced. The role of the analyst has changed but remains vital. However this fact has been recognised only slowly and is not adequately documented. The role of the laboratory manager is to understand the real concern of the analyst and to ease him in to his new function. Essentially this is a chemical systems role, applying his analytical chemical knowledge to develop and advance automatic analysis from a chemical rather than instrumental standpoint. The manager must help the analyst overcome his reluctance to understand the way the analyzer functions and to apply his chemical skills to exploit its potential. However complex and sophisticated the analyzer, its performance is related to the chemistry involved. The experienced analyst has an ongoing role in devising and adapting alternative methods which utilize more effectively the analyzer's capability to bring economic and scientific benefits to the laboratory through developing new, more elegant, methods, for future automated use. Instrument designers normally base their designs on well tried chemical methods and there is no guarantee that they are optimized for automated operation. It is the chemical analyst, in his new role as 'chemical systems analyst' who is required to provide the chemical input; nobody else is better qualified to do so.

**Table 3. Components of a complete analysis**

| |
|---|
| Sampling |
| Recording of sample information |
| Analysis |
| Calculation of results |
| Reporting and archiving of results |

The laboratory manager must all appreciate the problems of his junior staff. Initially they may neither understand the instrument nor the chemistry and whilst button-pushing and knob-twiddling are easily learned, any initial satisfaction gained can be quickly lost. It is for the manager to provide sympathetic in-house training at the right intellectual level, to encourage further formal education and, at the appropriate time, to arrange education and training in automatic analysis. This can take several forms; a number of colleges are now equipped for basic formal teaching in the subject and professional bodies offer specialist courses. Indeed, at the more advanced level several MSc courses in analytical chemistry or instrumental analysis offer an in-depth study of the subject. Attending a well chosen course at the right time in the staff's development can not only add to their knowledge and skills but can also engender a degree of self-confidence. This is one of the most important weapons in tackling new techniques and problems.

### Conclusion

The most significant problem for the laboratory manager is his own knowledge of, and his attitude to, automatic analysis. To launch a laboratory into an automatic regime successfully is a considerable achievement, involving, as has been summarized above, a breadth of knowledge and a fresh approach. Above all, he must stay abreast of new developments in automation. Much progress remains to be made in increasing the amount of meaningful information obtained from a sample. Further developments in automation technology, if correctly oriented, are a powerful way of continuing this process and the laboratory manager's role in it can be a challenging and rewarding one.

REFERENCES

[1] Stockwell, P. B. and Foreman, J.. K. 'Topics in Automatic Analysis, Vol. 1 (Ed. Foreman, J. K. and Stockwell, P.B.) Ellis Horwood, Chichester 1979 pp 30-33.
[2] Porter, D. G. and Stockwell, P. B. ibid pp 45-46 and 66-67.
[3] Foreman, J. K. and Stockwell, P. B. "Automatic Chemical Analysis", Ellis Horwood, Chichester 1975, pp 2-7.
[4] Ref 2 pp 49-66.
[5] Ref 3 pp 9-11.

